# Plastic‐Microbial BioRemediation DB: A Curated Database for Multi‐Omics Applications

**DOI:** 10.1111/1758-2229.70178

**Published:** 2025-09-08

**Authors:** Silvia Petraro, Chiara Tarracchini, Leonardo Mancabelli, Gabriele Andrea Lugli, Francesca Turroni, Marco Ventura, Christian Milani

**Affiliations:** ^1^ Laboratory of Probiogenomics, Department of Chemistry, Life Sciences, and Environmental Sustainability University of Parma Parma Italy; ^2^ Microbiome Research Hub University of Parma Parma Italy; ^3^ Department of Medicine and Surgery University of Parma Parma Italy

**Keywords:** bioremediation, database, microbial biodiversity, plastic biodegradation, plastic pollution

## Abstract

Plastic pollution is a major environmental challenge, with millions of tonnes produced annually and accumulating in ecosystems, causing long‐term harm. Conventional disposal methods, such as landfilling and incineration, are often inadequate, emphasising the need for sustainable solutions like bioremediation. However, the bacterial biodiversity involved in plastic biodegradation remains poorly understood. To address this gap, we present the *Plastic‐Microbial BioRemediation* (*Plastic‐MBR*) database, a curated multi‐omics resource that integrates publicly available genetic and enzymatic data related to putative plastic‐degrading microorganisms. This database supports *in silico* analyses of metagenomic data from plastic‐contaminated environments and comparative genomics, aiming to identify microbial taxa with potential plastic‐degrading functions. We validated the functionality of the *Plastic‐MBR* database by applying it to metagenomic datasets from plastic‐contaminated soil and river water, successfully identifying numerous putative plastic‐degrading genes across diverse microbial taxa. These results support the use of the *Plastic‐MBR* database as a tool to identify candidate bacteria for future experimental validation, strain isolation, and functional studies, ultimately contributing to a deeper understanding of microbial potential in plastic bioremediation. While this study focuses on database development and computational validation, future studies will be essential to confirm and translate these genomic predictions into effective bioremediation strategies.

## Graphical abstract

This study presents a novel multi‐omics framework leveraging the novel *Plastic‐Microbial BioRemediation* (*Plastic‐MBR*) database to map the taxonomic distribution of microbial enzymes involved in plastic degradation. Our approach, integrating metagenomic and *in silico* genomic analyses, enabled the identification of bacterial taxa, including previously unreported species, with potential plastic bioremediation capabilities.

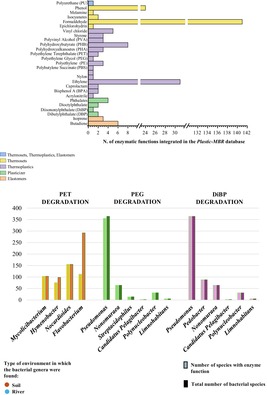

## 1 Introduction

Plastic is a significant environmental challenge, with millions of tonnes produced each year accumulating in ecosystems, causing damage due to its resistance to degradation and persistence. Conventional waste management solutions, such as incineration and recycling, often release pollutants or transfer plastics to other ecosystems (Briggs 2003; Geyer et al. 2017; Stubbins et al. 2021; Idumah and Nwuzor 2019; Chigwada and Tekere 2023; Amobonye et al. 2021).

Over the past decade, microbiological studies in plastic polluted environments, such as landfills, seas, and industrial wastewater, have identified microorganisms capable of degrading synthetic polymers. For example, *Ideonella sakaiensis* utilises PETase and MHETase to break down polyethylene terephthalate (PET) (Yoshida et al. 2016). Remarkably, genes encoding plastic‐degrading (PD) enzymes, such as cutinases, esterases, laccases, and other hydrolases, are present in phylogenetically diverse taxa isolated from contaminated environments, suggesting horizontal gene transfer (HGT) as a key mechanism for their spread. Recent bacterial genomic analyses have shown that HGT facilitates the transfer of adaptive biodegradation functions under environmental selection pressure (Bulka et al. 2024). These enzymes demonstrate remarkable versatility, degrading not only natural polymers such as lignin and cutin but also synthetic plastics like PET, polyurethane (PU), and polylactic acid (PLA). Additionally, bacteria such as *Pseudomonas putida* have been demonstrated to metabolise aromatic hydrocarbons through specialised enzymatic pathways, integrating breakdown products into central metabolism for energy production and biomass generation (Brzeszcz et al. 2023).

Thanks to this growing body of scientific literature on this topic, bioremediation has emerged as a promising biotechnological alternative for plastic waste remediation, exploiting microorganisms, fungi, and plants that produce PD enzymes to reduce their environmental impact (Lokesh et al. 2023; Borthakur et al. 2022; Azubuike et al. 2016; Amobonye et al. 2021).

However, research remains focused on a limited number of bacterial species, leaving gaps in our understanding of microbial diversity and genetic mechanisms involved in plastic degradation (Lokesh et al. 2023). This gap of knowledge restricts the scalability of bioremediation, highlighting the need for broader exploration of microbial diversity to develop effective strategies for different environments and types of plastic (Lokesh et al. 2023).

To address these gaps, this study introduces a multi‐omics approach incorporating the *Plastic‐Microbial BioRemediation* (*Plastic‐MBR*) database, which is a novel and comprehensive resource that integrates available genetic and enzymatic data on bacterial biodegradation of plastics, focusing on putative PD genes and enzymes identified through computational predictions and curated from public databases and literature. The *Plastic‐MBR* database has been designed as a strategic tool for *in silico* screening of isolated microbial genomes and complex metagenomic datasets, facilitating the identification and prioritisation of bacterial taxa with potential for plastic degradation. Additionally, it provides a valuable overview of the distribution of the genetic potential for plastic degradation across diverse environmental samples, enabling insights into how these functions are spread in different ecosystems.

This work focuses on the development and computational validation of the database, performing metagenomic and genomic analyses to demonstrate its utility. This approach provides a fundamental tool for future studies aimed at targeted cultivation, functional validation, and the development of applied bioremediation solutions.

## 2 Results and Discussion

### 2.1 Bioremediation of Plastics: Development of the *Plastic‐MBR* Database

**FIGURE 1:**
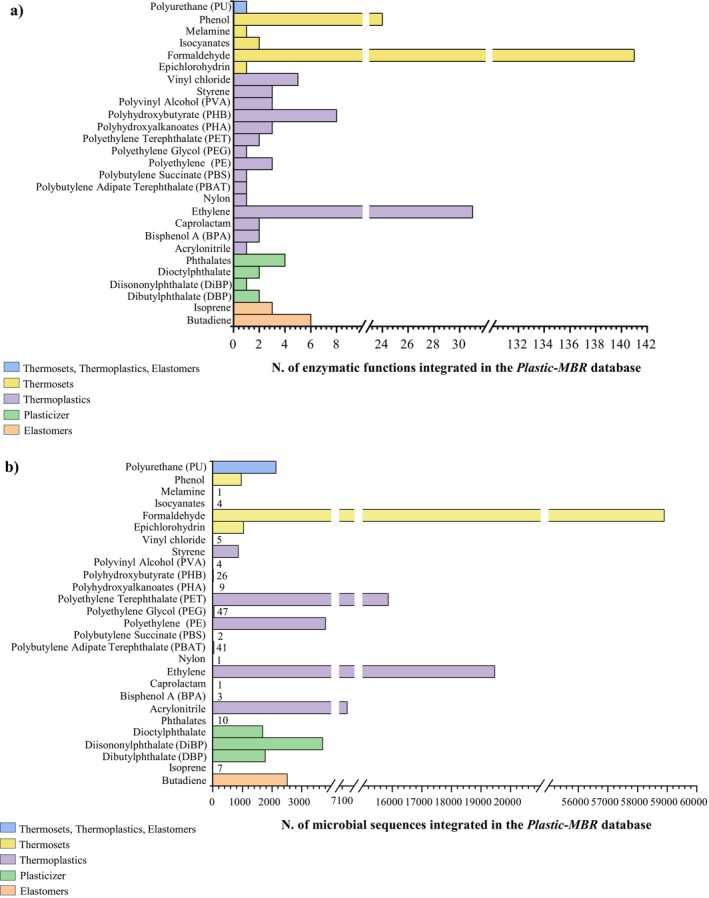
Structural overview of the *Plastic‐MBR* database. (a) The number of microbial plastic‐degrading enzymatic functions integrated in the Plastic‐MBR database. The data reflect a curated selection based on domain analysis, in which only non‐redundant functions with conserved and functionally relevant protein domains (as identified by InterProScan) were included. (b) The number of homologs and non‐homologs microbial sequences included in the database targeting each plastic compound. Columns colours indicate the subclass of each plastic compound.

Three primary subclasses of plastics, that is, thermoplastics, thermosets and elastomers, were identified through a comprehensive scientific literature survey based on their chemical structure and processing behaviour (Millican and Agarwal 2021; Varnava and Patrickios 2021). The class of plasticizers, that is, chemicals added to polymeric materials to improve their flexibility, malleability and durability (Marturano et al. 2017), was included due to its members showing ubiquitous presence in plastic formulations and well‐documented release into the environment, where they act as toxic and persistent pollutants and, for this reason, their microbial degradation represents a critical component of holistic plastic bioremediation strategies. Individual plastic compounds belonging to each subclass were systematically catalogued in 27 plastic compounds, including 15 thermoplastic compounds, five thermosets, two elastomers and four plasticizers (Figure 1A). Among the selected plastic compounds, PU is distinguished by its unique ability to belong to all three subclasses, depending on the chemical precursors used in its synthesis and the specific production process adopted (Figure 1A) (Rostampour et al. 2024).

To build the *Plastic‐MBR* database, we retrieved all publicly available bacterial amino acid sequences that were either annotated or computationally predicted to be hypothetically involved in the degradation of these plastic compounds. Data sources included specialised repositories such as the Plastic Biodegradation Database (PlasticDB) (Gambarini et al. 2022), the Plastics‐Active Enzymes Database (PAZy) (Buchholz et al. 2022), and the Plastic Microbial Biodegradation Database (PMBD) (Gan and Zhang 2019), while others were more generic, such as the curated enzyme database RHEA (Bansal et al. 2022). To improve the accuracy and breadth of the database, putative homologous sequences of known PD enzymes were also incorporated. Moreover, for 15 plastic pollutants, we were able to collect from 2 to 113 non‐homologous gene sequences (Table S3), supposed to encode for enzymes acting on different molecular chemical bonds, thus ensuring that the database can cover the currently known microbial metabolic versatility. This led to the development of the *Plastic‐MBR* database (http://probiogenomics.unipr.it/cmu/), which includes a total of 120,224 bacterial genes predicted to be putatively involved in the degradation of the 27 identified plastic matrices (Figure 1B; Table S1). Specifically, 47,410 enzyme sequences were putatively involved in the degradation of thermoplastics, 2535 enzymes were predicted to metabolise elastomers, 7206 enzymes were probably involved in the degradation of plasticizers, and 60,926 enzymes were predicted to target thermosetting compounds (Figure 1B; Table S2). In addition to these, 2147 sequences were predicted to be presumably associated with PU degradation, spanning the thermoset, thermoplastic, and elastomer categories (Figure 1B; Table S2).

Although these functional predictions are based on computational inference and require experimental validation, the *Plastic‐MBR* database represents a valuable starting point for identifying bacterial taxa and enzymatic functions with potential roles in plastic degradation. Its structure and content are designed to support downstream efforts in strain selection, genome mining, and in vitro characterisation for environmental bioremediation applications.

### 2.2 Validation of the *Plastic‐MBR* Database for Metagenomics Applications

To validate the predictive power of the *Plastic‐MBR* database, we applied it to functionally profile test cases corresponding to publicly available metagenomic datasets from plastic‐contaminated environments. Based on the premise that bacteria inhabiting plastic‐contaminated environments may have evolved or horizontally acquired the genetic potential to metabolise these materials, we analysed 212 public metagenomic samples from plastic‐contaminated soil (Wang et al. 2024; Schaerer et al. 2023; He et al. 2024) and 113 from river water samples (Zhou et al. 2022; Li et al. 2022) (Table S4), aiming to reconstruct the distribution of bacterial PD functions within these ecosystems (Milani et al. 2021).

For each of the identified PD functions, a Functional Plastic Degradation Index (FPDI) was calculated as the product of its prevalence (i.e., percentage of environmental samples showing that function) and its average relative abundance in the corresponding environment (water or soil) (formula: prevalence * average relative abundance) (Tables S5 and S6). This index provides a quantitative, metagenomics‐based estimate of the ecological significance and potential impact of each function in plastic degradation within its environmental context. Subsequently, to focus on the most relevant PD functions, we identified those falling in the 85th percentile of the FPDI, representing those with a higher impact in the degradation of plastic pollutants in the considered environments (Tables S5 and S6).

Notably, 23 PD functions showed an FPDI above the percentile threshold (FPDI > 0.0063308) and were putatively involved in the degradation of 12 of the 27 plastic compounds included in the *Plastic‐MBR* database (Table S5). In detail, seven are probably associated with the degradation of thermoplastics (polyethylene glycol [PEG], polyhydroxyalkanoate [PHA], ethylene, polyethylene [PE] acrylonitrile, PET and styrene), three in that of thermosetting compounds (formaldehyde, epichlorohydrin and phenol) and two functions in that of plasticisers (diisononyl phthalate [DiBP] and dibutyl phthalate [DBP]). Formaldehyde is widely used in the production of various resins commonly employed in thermosetting plastics. However, it is often released into the environment during plastic degradation or waste processing and represents toxic and persistent pollutants. For these reasons, its microbial degradation is considered a relevant target in bioremediation strategies aimed at mitigating the broader environmental impact of plastic waste.

Analogously, 24 bacterial PD functions identified in the contaminated river‐related metagenomic dataset showed a FPDI above the percentile threshold (FPDI > 0.0056725), potentially contributing to the degradation of 13 plastic compounds listed in the *Plastic‐MBR* database (Table S6). Specifically, these enzymes were predicted to be potentially associated with the degradation of three plasticizers (DiBP, DBP, Dioctylphthalate), seven thermoplastics (PEG, ethylene, PE, PHA, Polyethylene PET, acrylonitrile, polyhydroxybutyrate [PHB]) and three thermosets (formaldehyde, epichlorohydrin, phenol).

These results validated that the *Plastic‐MBR* database allows tracing in metagenomics datasets of several metabolic functions broadly represented across microbial communities from different polluted environments. While these findings do not imply direct in situ plastic degradation and just represent test cases showing case‐specific applications of the proposed database, they support the relevance of the *Plastic‐MBR* database as a screening tool for identifying microbial communities that may harbour genes with potential roles in bioremediation.

### 2.3 Validation of the *Plastic‐MBR* Database for Screening of Key Microbial Taxa Involved in Plastic Bioremediation

**FIGURE 2:**
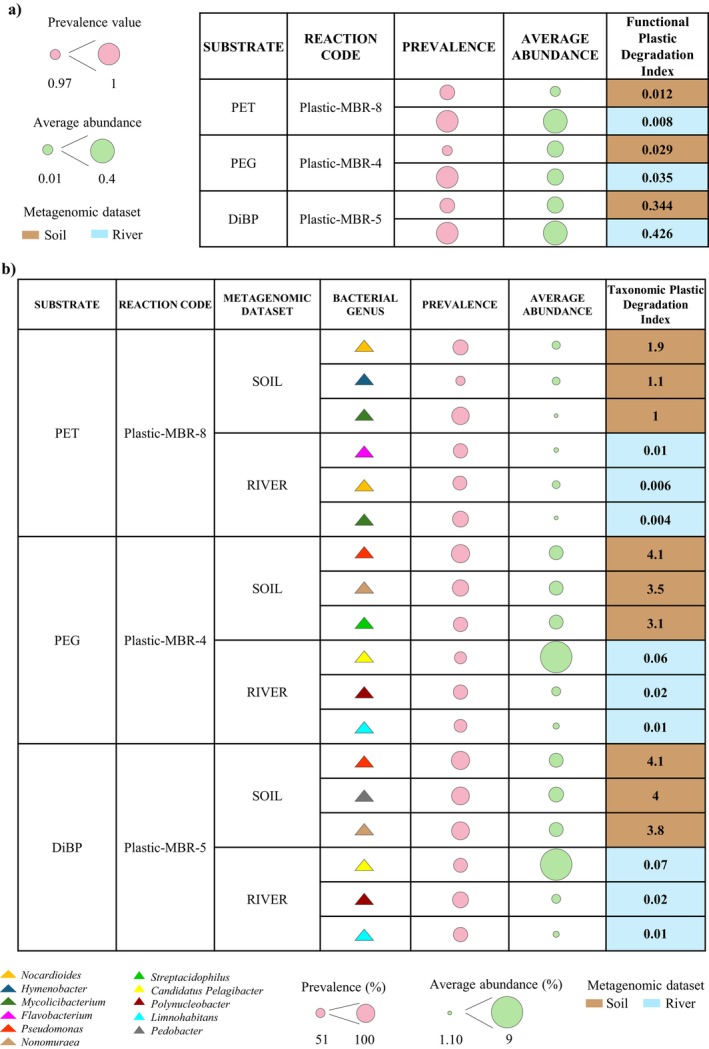
Calculation of the Plastic Degradation Indices (FPDI and TPDI) in metagenomic samples. (a) The calculation of the Plastic Functional Degradation Index (FPDI) by integrating the prevalence (pink circles) and relative abundance (green circles) of bacterial functions potentially involved in the degradation of PET, PEG and DiBP in plastic‐contaminated river water and soil metagenomic samples. (b) The calculation of Taxonomic Plastic Degradation Index (TPDI) through the integration of the prevalence (pink circles) and metagenomic relative abundance (green circles) of microbial taxa associated with the degradation of PET, PEG and DiBP into the plastic‐contaminated river water and soil metagenomic samples.

To strengthen the proof‐of‐concept application of the *Plastic*‐*MBR* database and establish associations between predicted bioremediation capabilities and microbial taxa, metagenomic reads mapped to PD functions were further analysed through taxonomic classification. This approach allowed us to generate taxonomic profiles of bacteria predicted to contribute to each identified PD function, providing insights into the microbial players potentially involved in plastic degradation within the sampled environments. Remarkably, these associations are based solely on *in silico* predictions, and thus require further experimental validation to confirm their actual biodegradative roles. Nonetheless, these predictions offer a valuable framework for prioritising microbial candidates in future functional studies. Specifically, among the PD functions identified in the metagenomic datasets, those corresponding to degradation activities against PET (Plastic‐MBR‐8), PEG (Plastic‐MBR‐4), and DiBP (Plastic‐MBR‐5) were selected as test cases due to their FPDI value above the percentile threshold for each of the chosen enzyme functions (Figure 2A), as well as their wide distribution and significant ecological impact on environmental pollution (Umdagas et al. 2025; Muneeswaran et al. 2025; Borch et al. 2006). In particular, PET is one of the world's most widely used plastic polymers, with an annual production exceeding 70 million tons (Zhang et al. 2025), while dispersants such as PEG are used as non‐ionic surfactants in biocide and household products to improve solubility and efficacy (Muneeswaran et al. 2025). Additionally, DiBP, a plasticiser mainly used in PVC‐based products, is recognised for its toxic properties, posing a risk to both aquatic and terrestrial fauna (Borch et al. 2006; Chen et al. 2020).

As expected for environmental samples, despite the use of an updated database of reference genomes (updated January 2025), most taxonomical profiles were classified down to the genus level, likely due to the presence of not‐yet classified species. However, similarly to the approach used for functional profiling, a Taxonomic Plastic Degradation Index (TPDI) was computed for each microbial taxa profiled within each of the three selected PD activities. This index integrates the prevalence of putative degrading microbial taxa and their average relative abundance in the corresponding environment (water or soil) (formula: prevalence * average relative abundance), providing a comprehensive measure of their ecological relevance in plastic degradation (Figure 2B). Therefore, by focusing on the three microbial genera with the highest TPDI values for each PD function (in both environments), we identified the main microbial taxa potentially involved in the degradation of PET, PEG, and DiBP in the examined environments. In the case of plastic‐contaminated soil samples (Table S7), the genera *Nocardioides*, *Hymenobacter*, and *Mycolicibacterium* showed the highest TPDI values in PET degradation (TPDI index corresponding to 1.9, 1.1 and 1, respectively), while genera *Pseudomonas*, *Nonomuraea*, and *Streptacidiphilus* showed a notable TPDI score for PEG degradation (4.1, 3.5, and 3.1, respectively). In addition, the genera *Pseudomonas*, *Pedobacter*, and *Nonomuraea* showed the highest TPDI values in DiBP degradation (TPDI index corresponding to 4.1, 4 and 3.8, respectively).

Similarly, in plastic‐contaminated river samples (Table S8), the genera *Flavobacterium*, *Nocardioides*, and *Mycolicibacterium* showed the highest TPDI values in PET degradation (TPDI index corresponding to 0.01, 0.006 and 0.004, respectively). The genera *Candidatus Pelagibacter*, *Polynucleobacter* and *Limnohabitans* were among the three taxa with the highest TPDI scores in the degradation of PEG (0.06, 0.02 and 0.01, respectively) and DiBP (0.07, 0.02 and 0.01, respectively).

To validate our approach, we performed a literature investigation aimed at investigating if the above‐mentioned bacterial genera were already reported as plastic‐degraders. Interestingly, members of the *Pseudomonas* and *Mycolicibacterium* genera were already known for their ability to degrade long‐chain hydrocarbons and other pollutants, including nornicotine and polychlorinated biphenyls (Wang et al. 2025; Dang et al. 2023; Šrédlová and Cajthaml 2022). Remarkably, this observation demonstrates the reliability and efficacy of our approach in identifying microbial sequences associated with plastic bioremediation capabilities. However, we also identified taxa that had not previously been associated with the degradation of these compounds, such as *Hymenobacter*, *Nonomuraea*, *Streptacidiphilus*, *Pedobacter*, *Polynucleobacter* and *Limnohabitans*. This highlights the potential of our database to uncover novel taxa with promising bioremediation capabilities, which could play a key role in the microbial breakdown of plastic pollutants.

Overall, these findings validated that the *Plastic‐MBR* database also allows revealing distinct bacterial assemblages involved in plastic degradation in metagenomic samples. Thus, allowing exploration of how different microbial consortia driving this process depend on the environmental context. This is now a key research target for the future development of selected bacterial consortia, adapted to the specific characteristics of each environment and representing a promising solution for managing plastic contaminants in a more targeted and sustainable manner.

### 2.4 Validation of the *Plastic‐MBR* Database for Genomic Investigations and Selection of Promising Species for Plastic Bioremediation

To further support the proof‐of‐concept application of the *Plastic*‐*MBR* database and since the taxonomic assignment in environmental samples is generally limited to the genus level, we performed an inter‐genus comparative genomics analysis to refine the association to the species level as a further test case application of the *Plastic‐MBR* database. Specifically, for the three bacterial genera that showed the highest TDPI index for PET, PEG, and DiBP degradation in soil and water river environments (Table S9), we retrieved reference genome sequences of known species from public databases. These genomes were then screened using our *Plastic‐MBR* database to identify genes potentially involved in the degradation of plastic compounds, enhancing the functional predictions and providing more precise insights into the potential contributions of specific microbial species to plastic biodegradation.

**FIGURE 3:**
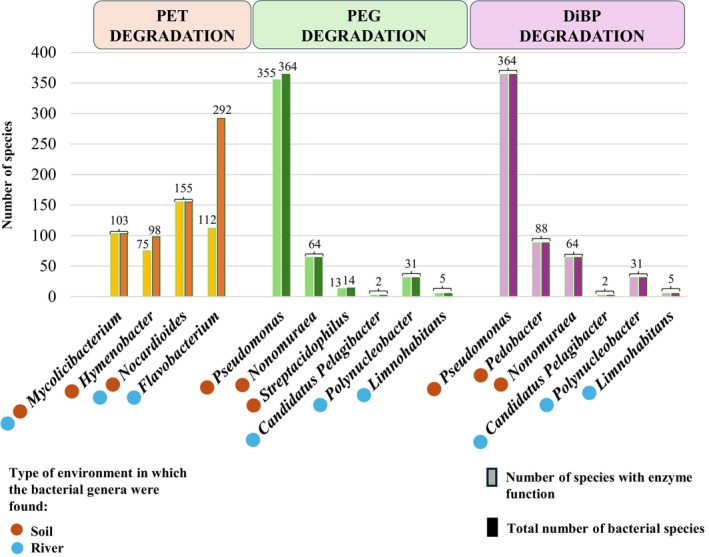
Distribution of enzymatic potential for biodegradation of synthetic polymers and plasticisers across bacterial species. The light green bars represent the number of species of each bacterial genus for which the reference genomes showed the presence of PD functions for PET, PEG and DiBP degradation. In contrast, the dark green bars represent the number of species of each bacterial genus. The brown circles represent the reference strains of each genus identified in the soil metagenomic datasets, while the blue circles represent the reference strains of each genus identified in the river water sample datasets.

Regarding the soil environment, our results showed that all species from the *Mycolicibacterium* and *Nocardioides* genera, as well as 77% of *Hymenobacter*, harboured the genetic potential to encode a cutinase presumably involved in PET degradation (Figure 3; Tables S10 and S11). Similarly, 98%, 100% and 93% of species from *Pseudomonas*, *Nonomuraea*, and *Streptacidiphilus* genera, respectively, appeared to possess the enzyme sequences for polyethylene glycol dehydrogenase, potentially contributing to PEG degradation (Figure 3; Tables S10 and S12). Additionally, all members of the *Pseudomonas*, *Pedobacter*, and *Nonomuraea* genera exhibited the genetic potential for DiBP degradation (Tables S10 and S13).

In the contaminated aquatic environment, in addition to all members of the *Mycolicibacterium* and *Nocardioides* genera already reported above for the soil, 38% of the species from the *Flavobacterium* genus showed enzyme functions potentially involved in PET degradation (Figure 3; Tables S10 and S11). Regarding PEG degradation, both known members of the *Candidatus Pelagibacter* taxon were predicted to possess the enzyme sequence for polyethylene glycol dehydrogenase, a feature also identified in all species belonging to the *Polynucleobacter* and *Limnohabitans* genera (Figure 3; Tables S10 and S12). Moreover, all members of the *Candidatus Pelagibacter*, *Polynucleobacter*, and *Limnohabitans* taxa also showed the genetic potential for DiBP degradation (Figure 3; Tables S10 and S13).

While these bioinformatic analyses expand our understanding of microbial potential, experimental validation remains critical to confirm the actual PD activity of these species.

Noteworthy, some of the species identified as possessing the genetic arsenal for plastic degradation have already been recognised for their potential role in environmental bioremediation (Zhu et al. 2024; Bhattacharyya et al. 2023), further validating the reliability of our approach at the species level. Specifically, *Pseudomonas stutzeri* is the only species currently known to degrade PEG (Obradors and Aguilar 1991), while other *Pseudomonas* species are well‐documented for their ability to metabolise various environmental pollutants, including polycyclic aromatic hydrocarbons, toluene, cyanide, heavy metals, and pesticides (Vélez et al. 2021; Medić and Karadžić 2022; Balíková et al. 2022; Hu et al. 2023; Wasi et al. 2013; Di Martino et al. 2012). However, their potential role in PEG or DiBP degradation had not been previously recognised, as evidenced by our *in silico* findings. Similarly, although members of the *Hymenobacter* and *Flavobacterium* genera have been shown to degrade various environmental contaminants, including aromatic compounds and pesticides (Guo et al. 2020; Ortiz‐Hernández et al. 2003; Sun et al. 2011), no known species from these genera have, to date, been reported to degrade PET. Additionally, while *Pedobacter* species have been associated with the degradation of certain environmental pollutants, including polycyclic aromatic hydrocarbons (Mao et al. 2012; Breton‐Deval et al. 2020; Chang et al. 2017), their involvement in DiBP breakdown remains uncharacterised, pointing to their potential ecological role in pollutant biotransformation.

These test‐case genomic analyses further validated the *Plastic*‐*MBR* database as a valuable tool for identifying previously unrecognised microbial species with potential PD capabilities, which can now be prioritised for experimental in vitro validation.

## 3 Conclusion

The identification of efficient PD microorganisms and their associated enzymatic pathways remains challenging when relying solely on conventional microbiological approaches (Varnava and Patrickios 2021; Coudert et al. 2023). To address this gap, we established the *Plastic‐MBR* database, which is a curated multi‐omics resource integrating publicly available genetic and enzymatic data related to presumably PD microorganisms. Designed as a strategic tool for *in silico* analyses, *Plastic*‐*MBR* enables the screening of both isolated microbial genomes and complex metagenomic datasets, facilitating the identification and prioritisation of bacterial taxa with potential PD functions.

The database also provides a valuable overview of the distribution of plastic‐degradation potential across environmental microbial communities, offering insights into how these functions are disseminated in diverse ecosystems. This is particularly relevant for studying complex environments through metagenomic screening, allowing researchers to trace functional capabilities at the community level and highlight specific taxa for further investigation.

The functionality of the *Plastic‐MBR* database was validated through a set of test cases, including metagenomic datasets from plastic‐contaminated soil and river water and subsequent targeted comparative genomics investigations. Results revealed how the proposed database allows the identification of numerous putative PD genes across diverse microbial taxa, including candidate species not previously associated with plastic degradation. Importantly, these are computational predictions based on sequence homology and genomic context, and as such, they require experimental confirmation to verify the actual biodegradative activity of the genes or taxa involved. These analyses serve as an *in silico* validation of the database and provide a framework to prioritise bacterial candidates for future experimental validation, strain isolation, functional studies, and metagenomic screening of microbial populations across diverse environmental matrices.

Moreover, although no clear phylogenetic patterns emerged correlating taxonomy with PD functions, the genetic variability included in the *Plastic‐MBR* database also allowed observation of the widespread occurrence of relevant genes among taxonomically diverse bacteria, which supports a significant role for HGT in disseminating these functions (French et al. 2020).

Overall, this study establishes the *Plastic‐MBR* database as a valuable resource for guiding further investigations into microbial plastic biodegradation. The integration of computational predictions with targeted experimental approaches will be essential to elucidate molecular mechanisms and to design environment‐specific microbial consortia for sustainable bioremediation since these efforts could ultimately facilitate the development of scalable biotechnological solutions to mitigate plastic pollution in diverse ecosystems.

Future refinements of the *Plastic*‐*MBR* database may include a clearer separation between genes related to the degradation of small and easily biodegradable molecules, such as formaldehyde, and those involved in breaking down more complex plastic polymers. Although these simpler compounds are relevant due to their presence in plastic‐related pollution, managing them as a separate category in future releases could improve the accuracy of functional predictions. In addition, the database will be expanded with the integration of data on non‐coding RNAs involved in the regulation of biodegradation, synthetic metabolic pathways designed to broaden the spectrum of treatable contaminants, and artificial intelligence techniques for the automatic prediction of new enzymatic functions and the functional annotation of uncharacterised sequences. These developments will make *Plastic‐MBR* not only a database, but a dynamic platform supporting systemic research on a global scale, which is essential to address future environmental challenges.

## 4 Materials and Methods

### 4.1 Construction of the *Plastic‐MBR* Database

The *Plastic‐MBR* database was developed using publicly available amino acid sequences corresponding to bacterial enzymes involved in the bioremediation of various plastic pollutants, including thermoplastics, thermosets, and elastomers (Viglioli et al. 2024).

In detail, for each major plastic category, we identified the most environmentally prevalent plastic polymers and retrieved bacterial enzyme sequences with experimentally validated or predicted degrading capabilities from publicly accessible databases. These include databases specific to the degradation of plastic pollutants, such as the PlasticDB (Gambarini et al. 2022), PAZy (Buchholz et al. 2022), and PMBD (Gan and Zhang 2019), but also more generic databases, such as the Rhea‐Annotated Reactions Database (Rhea database) (Bansal et al. 2022).

For each identified enzyme sequence, the Rhea database links to the corresponding UniProt (Coudert et al. 2023), where all homologous sequences related to that enzymatic function are deposited.

Additionally, for functions identified in databases specific to plastic compounds, homologous sequences were retrieved from the RefSeq database.

Protein domain analysis using InterProScan 5.68–100.0 (Quevillon et al. 2005) was employed both to confirm homology and to perform stringent filtering aimed at avoiding overestimation and redundancy, by retaining only non‐redundant enzymatic functions supported by conserved and functionally relevant protein domains. The inclusion threshold was based on the presence of specific conserved protein domains characteristic of enzymatic functions with experimentally validated activity, as supported by reference sequences from public databases.

### 4.2 Metagenomic Analysis of Soil and River Samples

Datasets corresponding to shotgun metagenomic samples of plastic‐contaminated soil and river water were downloaded via the NCBI SRA repository. In detail, the obtained fastq were quality‐filtered using the fastq‐mcf software (mean quality score > 20 and read length > 100 bp; Viglioli et al. 2024). Subsequently, METAnnotatorX2 was used to obtain taxonomic and functional profiles from the metagenomic datasets (Li et al. 2022). In particular, for the study, the *Plastic‐MBR* database was integrated into METAnnotatorX2, allowing the precise identification of bacterial PD functions. This analysis was applied to the metagenomic datasets with the following parameters: 5,000,000 reads for metabolic assignment (−query‐cover 80, −evalue 1 × 10^−8^, and −max‐target‐seqs 1) (Milani et al. 2025). In addition, METAnnotatorX2 software was also used to obtain taxonomic profiles of the reads pool associated with PD function identified in the datasets.

### 4.3 Genomic Analysis

To identify promising bacterial species for the environmental bioremediation of plastic pollutants, a comparative genome analysis was performed on publicly available genome sequences from bacterial genera predicted to encode PD enzymes in our metagenomic analysis. Specifically, for each species, the genome sequence of reference bacterial strains was downloaded from the National Centre for Biotechnology Information (NCBI). A protein Basic Local Alignment Search Tool (BLASTp) analysis (cutoff e‐value of 1 × 10^−10^, at least 50% identity percentage) was then performed against the *Plastic‐MBR* database. To ensure consistent gene prediction and annotation, we first processed all the obtained genome sequences using Prokka v1.14.6 with default parameters (Seemann 2014).

### 4.4 Phylogenomic Analysis

To investigate the phylogenetic relationships among the genomes within PD genera, core sequences were identified with the Roary v3.13.0 software (Page et al. 2015; Seemann 2014). The phylogenetic tree was then constructed using raxmlHPC v8 (Stamatakis 2014) and visualised with iTOL v6.8.1 (Interactive Tree Of Life), an online tool for annotating, managing, and graphically representing phylogenetic trees.

## Author Contributions

**Silvia Petraro:** writing – original draft, formal analysis. **Chiara Tarracchini:** formal analysis, writing – original draft. **Leonardo Mancabelli:** data curation. **Gabriele Andrea Lugli:** data curation. **Francesca Turroni:** supervision, writing – review and editing. **Marco Ventura:** conceptualization, supervision, writing – review and editing. **Christian Milani:** conceptualization, supervision, writing – review and editing.

## Conflicts of Interest

The authors declare no conflicts of interest.

## Supporting information


**Table S1:** Bacterial amino acid sequences coding for enzymes involved in the degradation of plastic pollutants integrated in the plastic‐MBR database.
**Table S2:** Bacterial enzymes involved in the degradation of polluting compounds thermoplastics, thermosets, elastomers and plasticizers.
**Table S3:** Non‐homologous gene sequences integrated in the Plastic‐MBR database for 18 plastic pollutants.
**Table S4:** Metagenomic datasets from soil and river samples known to be contaminated with plastics analysed in the study.
**Table S5:** Calculation of the Functional Plastic Degradation Index (FPDI) for metagenomic datasets of soil samples contaminated with plastic pollutants.
**Table S6:** Calculation of the Functional Plastic Degradation Index (FPDI) for metagenomic datasets of river samples contaminated with plastic pollutants.
**Table S7:** Bacterial genera present in soil samples with the genetic potential to reduce the environmental bioavailability of PET, PEG and DiBP.
**Table S8:** Bacterial genera present in river samples with the genetic potential to reduce the environmental bioavailability of PET, PEG and DiBP.
**Table S9:** Reference genomes of the genera *Flavobacterium*, *Hymenobacter*, *Mycolicibacterium*, *Nocardioides*, *Candidatus Pelagibacter*, *Pedobacter* and *Pseudomonas* on which genomic analyses were performed.
**Table S10:** Functional analysis on the reference genomes of the bacterial genera *Mycolicibacterium*, *Hymenobacter*, *Nonomuraea* and *Streptacidophilus*.
**Table S11:** Blastp analysis on the reference genomes of the genera *Mycolicibacterium*, *Hymenobacter*, *Flavobacterium* and *Nocardioides* for the enzymatic.
**Table S12:** Blastp analysis on the reference genomes of the genera *Pseudomonas* and *Candidatus Pelagibacter* for the enzymatic function potentially involved in PEG degradation genome.
**Table S13:** Blastp analysis on the reference genomes of the genera *Pseudomonas* and *Candidatus Pelagibacter* for the enzymatic function potentially involved in DiBP degradation genome.

## Data Availability

The data that supports the findings of this study are available in the Supporting Information material of this article.
